# Improvement of exertional dyspnea and breathing pattern of inspiration to expiration after bronchial thermoplasty

**DOI:** 10.1186/s13223-018-0276-3

**Published:** 2018-10-29

**Authors:** Keisuke Miki, Mari Miki, Kenji Yoshimura, Kazuyuki Tsujino, Hiroyuki Kagawa, Yohei Oshitani, Yuko Ohara, Yuki Hosono, Ryuya Edahiro, Hiroyuki Kurebe, Seigo Kitada

**Affiliations:** Department of Respiratory Medicine, National Hospital Organization Toneyama National Hospital, 5-1-1 Toneyama, Toyonaka, Osaka 560-8552 Japan

**Keywords:** Asthma, Cardiopulmonary exercise testing, Dynamic hyperinflation, Ventilation

## Abstract

**Background:**

Bronchial thermoplasty (BT) is a bronchoscopic treatment that can ameliorate the symptoms of severe asthma. However, little is known about the mechanism by which BT improves exertional dyspnea without significantly changing the resting pulmonary function in asthmatics. To understand the mechanism, cardiopulmonary variables were investigated using cardiopulmonary exercise testing (CPET) in a patient with severe asthma before and after BT.

**Case presentation:**

A 57-year-old Japanese man visited our hospital for consultation of the intractable asthma, which we managed with three treatment sessions of BT. Comparison of the findings pre-BT and at 1 year after BT demonstrated that (1) the resting tests for respiration showed no improvement in forced expiratory volume in 1 s, but the forced oscillation technique showed decreases in both inhalation and exhalation respiratory resistance values, and (2) the CPET results showed (i) improvement in exertional dyspnea, exercise endurance, and arterial oxygen saturation at the end of exercise; (ii) that the expiratory tidal volume exceeded the inspiratory tidal volume during exercise, which implied that a sufficient exhalation enabled longer inspiratory time and adequate oxygen absorption; and (iii) that an increase in respiratory frequency could be prevented throughout exercise.

**Conclusions:**

This case report described a novel mechanism of BT in improving exertional dyspnea and exercise duration, which was brought about by ventilatory improvements related to the breathing pattern of inspiration to expiration.

## Case presentation

**Fig. 1:**
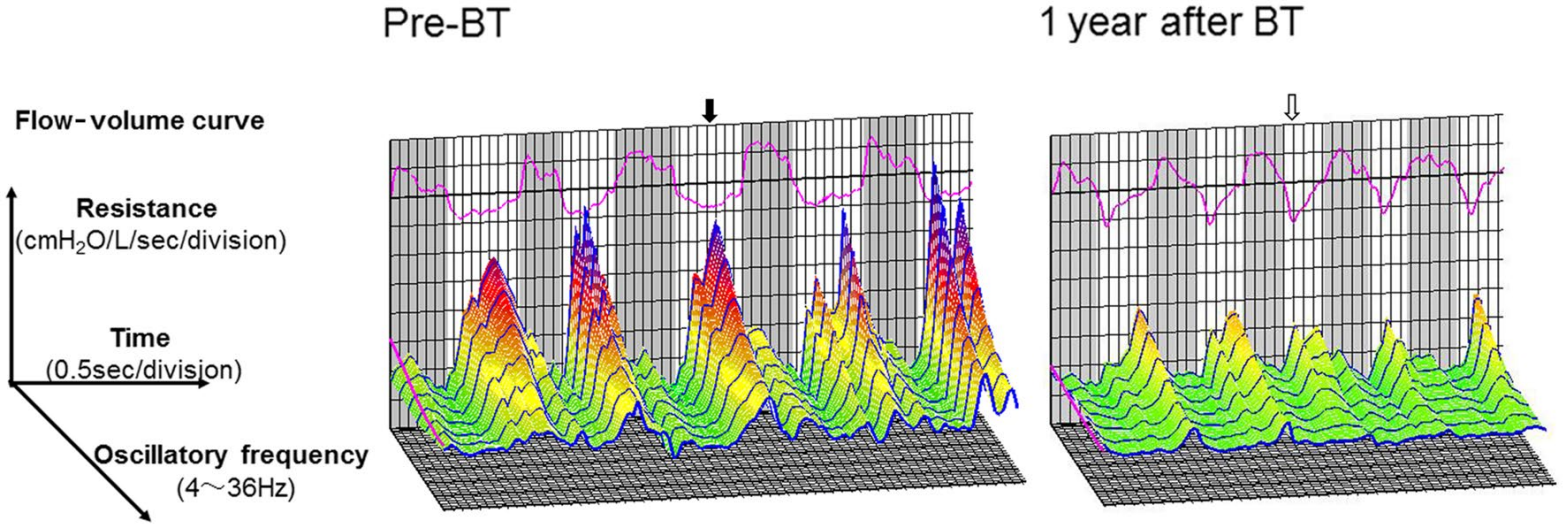
Changes in the resting respiratory system resistance on the flow-volume curve at pre-BT and at 1 year after BT. The forced oscillation technique was used. Pre-BT, a semicircular flow-volume curve was detected in the expiratory phase (white zone), with the nadir (closed arrow) detected in the middle of the phase. At 1 year after BT, the flow-volume curve was changed to a triangular shape, with the nadir (open arrow) detected in the early expiratory phase. The gray zone represents the inspiratory phase. *BT* bronchial thermoplasty

**Fig. 2:**
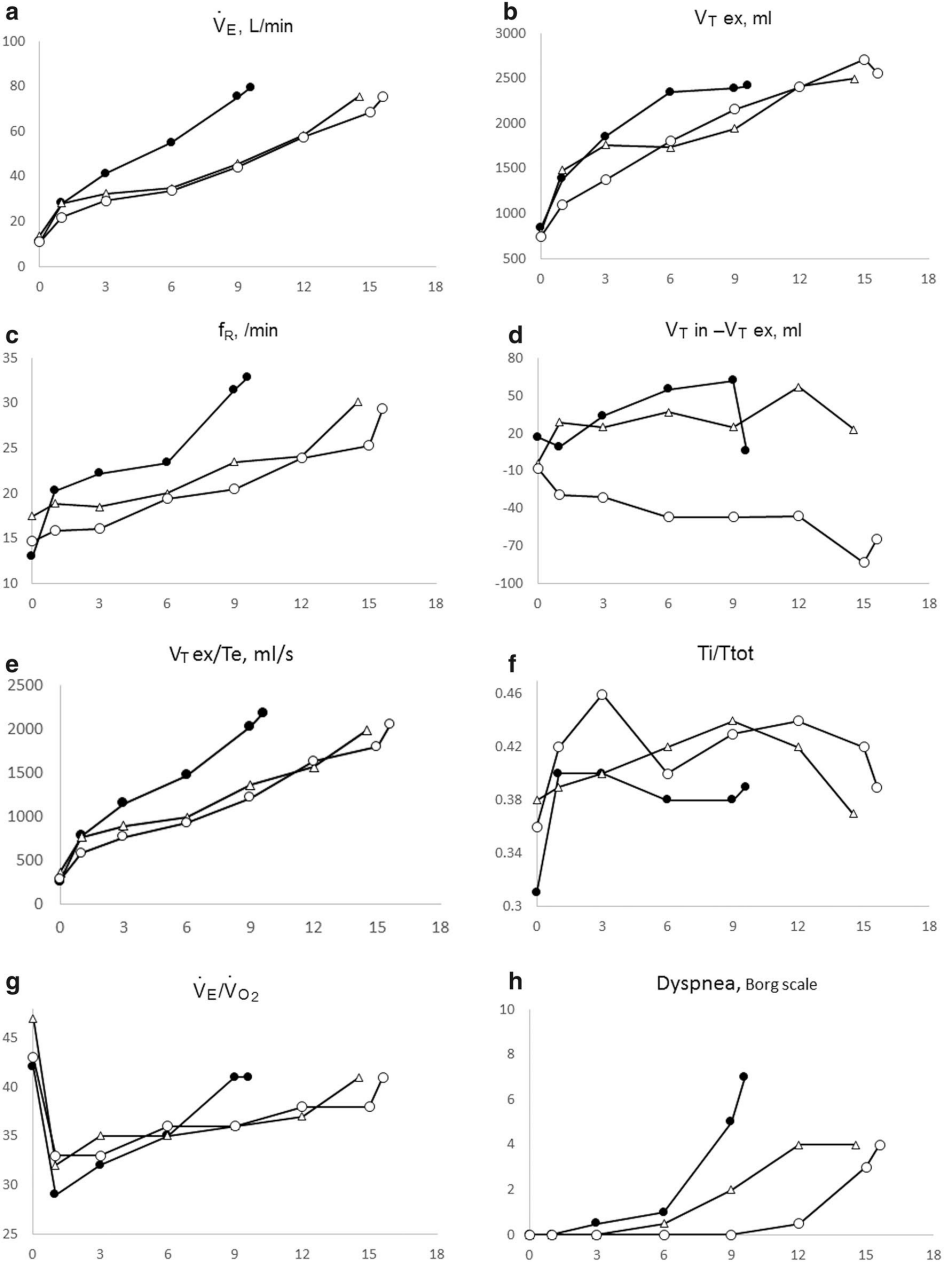
Changes in the cardiopulmonary variables before and after BT. Changes in the ventilatory variables at pre-BT and at 3 months and 1 year after BT. Cardiopulmonary function was assessed by three procedures of incremental cardiopulmonary exercise testing using a similar treadmill protocol. *BT* bronchial thermoplasty, *ex* expiratory, *f*_*R*_ breathing frequency, *in* inspiratory, *Te* expiratory time, *Ti/Ttot* the ratio of inspiratory time to total breathing cycle time, $\dot{V}_{E}$ minute ventilation, $\dot{V}_{{O_{2} }}$ oxygen uptake, *V*_*T*_ tidal volume. Closed circle: pre-BT; open triangle: 3 months after BT; open circle: 1 year after BT

A 57-year-old Japanese man visited our hospital for consultation of asthma attacks with exertional dyspnea. When he was about 30 years old, he was started on asthma treatment by a local physician. However, the asthma attacks occurred frequently despite triple therapy with high-dose inhaled corticosteroids, inhaled long-acting beta-2 agonist drugs, and long-acting anticholinergic drugs. He had no history of smoking. Blood test findings showed 7.1% eosinophilia (460/μL) and an elevated total IgE level at 256 IU/mL (specific IgE for house dust: 0.97 U_A_/mL; for mite: 1.18 U_A_/mL). There was bronchial wall thickening on both lungs on plain computed tomography of the chest. Exhaled nitric oxide concentration was increased at 68 ppb. After managing the asthma attack with oral intake of prednisolone at 30 mg/day for 6 days, there was persistence of dyspnea and fluctuations in forced expiratory volume in one second (FEV_1_) values from 1.17 L before steroid treatment to 2.33 L after steroid treatment. The patient was diagnosed as intractable asthma based on his history and the clinical course. Using the Alair Bronchial Thermoplasty (BT) System (Boston Scientific Corporation, MA, USA), BT was performed in three treatment sessions with a different region of the lung. Each treatment was performed approximately 3 weeks apart. Because the stenosis was observed in each lobe bronchus due to bronchial mucosal thickening, total sessions consisted of 98 activations. At 1 year after BT, the resting tests for respiration showed no improvement in FEV_1_, but the forced oscillation technique (FOT) [1, 2] (MostGraph, Chest M.I., Tokyo, Japan) showed decreases in both inhalation and exhalation respiratory resistance values (Table 1 and Fig. 1). Assessment of asthma control scores [3] showed improvement from 19 before BT to 25 at 1 year after BT. CPET (Aero monitor AE310S, Minato Medical Science Co., Ltd., Osaka, Japan) was performed using a similar treadmill protocol by Sheffield [4]. All the CPET results indicated that exercise was terminated when the target heart rate (THR), which was calculated as 220-age in years was reached; thereafter, the CPET results were evaluated (Table 2, Fig. 2). At the end of exercise, comparison of the findings at pre-BT and at 1 year after BT showed (1) improvement in dyspnea based on the Borg scale; (2) longer exercise time to reach the THR; and (3) increase in arterial oxygen saturation (SpO_2_).

**Table 1:** Changes in resting pulmonary function and forced oscillatory parameters after BT

	Pre-BT	3 months after BT	1 year after BT
Pulmonary function test			
FEV_1_, L	2.63	2.52	2.38
%FEV_1_, % predicted	76.2	74.3	70.8
FEV_1_/FVC, %	51.2	47.4	46.5
VC, L	5.10	5.21	5.17
IC, L	3.38	3.51	3.23
Forced oscillation technique			
R5 ex, cmH_2_O/L/s	3.83	3.65	2.42
R5 in, cmH_2_O/L/s	1.96	2.12	1.66
R20 ex, cmH_2_O/L/s	2.77	2.68	2.03
R20 in, cmH_2_O/L/s	1.80	2.12	1.68
Fres ex, Hz	9.48	10.56	8.07
Fres in, Hz	7.41	8.67	7.19

*BT* bronchial thermoplasty, *ex* expiratory, *FEV*_*1*_ forced expiratory volume in 1 s, *Fres* resonant frequency, *FVC* forced vital capacity, *IC* inspiratory capacity, *in* inspiratory, *R5* the resistance at 5 Hz, *R20* the resistance at 20 Hz, *VC* vital capacity

**Table 2:** Post-BT changes in cardiopulmonary function assessed at THR during CPET

	Pre-BT	3 months after BT	1 year after BT
Dyspnea, Borg scale	7	4	4
HR response, %	105	109	103
Exercise time, min	9.6	14.5	15.5
${\dot{\text{V}}}_{{{\text{O}}_{ 2} }}$, mL min^−1^ kg^−1^	26.0	25.7	25.0
${\dot{\text{V}}}_{\text{E}}$, L min^−1^	79.4	75.4	75.3
ΔFO_2_, %	2.97	3.09	3.04
V_Tex_, mL	2412	2495	2559
V_T_ in − V_Tex_, mL	6	23	− 64
f_R_, breaths min^−1^	32.9	30.2	29.4
Ti/Ttot	0.39	0.37	0.39
V_D_/V_Tex_	0.28	0.28	0.27
${\dot{\text{V}}}_{\text{E}} /{\dot{\text{V}}}_{{{\text{O}}_{ 2} }}$	41	41	41
${\dot{\text{V}}}_{\text{E}} /{\dot{\text{V}}}_{{{\text{CO}}_{ 2} }}$	38	35	36
SpO_2_, %	91	96	94
AT, mL min^−1^	1147	1277	1230

*AT* anaerobic threshold obtained by the V-slope method, *BT* bronchial thermoplasty, *CPET* cardiopulmonary function testing, *ΔFO*_*2*_ the inspired oxygen concentration (FiO_2_) minus the expired oxygen concentration (FeO_2_), *ex* expiratory, *f*_*R*_ breathing frequency, *HR* heart rate, *in* inspiratory, *SpO*_*2*_ oxygen saturation, *Ti/Ttot* the ratio of inspiratory time to total breathing cycle time, *THR* target heart rate = 220 − age (years), $\dot{V}_{{CO_{2} }}$ carbon dioxide output, *V*_*D*_*/V*_*Tex*_ physiologic dead space/tidal volume ratio, $\dot{V}_{E}$ minute ventilation, $\dot{V}_{{O_{2} }}$ oxygen uptake, *V*_*T*_ tidal volume

## Discussion

This case report described improvements in the exertional breathing pattern as the novel mechanism by which BT improved exertional dyspnea in a patient with intractable asthma. BT is a bronchoscopic treatment that can ameliorate the subjective symptoms of severe bronchial asthma that is difficult to control [5–9]. In the future, BT is expected to be one of the treatment strategies for severe asthma. However, the mechanisms by which BT improves the subjective symptoms of asthma without significantly changing the resting pulmonary function [6, 8] are yet to be elucidated. Exertional dyspnea is a common symptom in asthma, and the mechanisms of it in asthma are complex [10]. In the present case, we focused on the pattern of exertional ventilation because minute ventilation (${\dot{\text{V}}}_{\text{E}}$) decreased during exercise after BT (Fig. 2a). Although not all asthmatics develop dynamic hyperinflation (DH) [10], before BT in this case, the patient presented with DH during mid-exercise at pre-BT, because, based on the finding that the expiratory tidal volume (V_Tex_) reached a plateau followed by a sharp increase in respiratory frequency (f_R_). At 1 year after BT, the breathing pattern of DH improved (Fig. 2b, c). Similarly, a study by Thomen et al. [11] used a combination of helium 3, magnetic resonance imaging, and CT to demonstrate that after BT, the ventilation defects decreased with time.

Although improvement of DH was important, the reduced ${\dot{\text{V}}}_{\text{E}}$ requirement throughout exercise and the prolonged exercise time obtained in the present case were noteworthy (Fig. 2a and Table 2). Considering that both V_Tex_ and f_R_ during exercise were reduced after BT (Fig. 2b, c), exertional dyspnea, especially during mid-exercise, may have pathophysiologic mechanisms other than the occurrence of DH only in the late exercise phase. V_Tex_ exceeded inspiratory tidal volume (V_Tin_) form resting to peak exercise, especially at 1 year after BT (Fig. 2d). This implied that the patient could exhale sufficiently after BT, which improved both the static and dynamic hyperinflation throughout exercise. Furthermore, mean expiratory flow (V_Tex_/expiratory time: Te) was reduced throughout exercise (Fig. 2e). We deduced that the obtained ventilation pattern at 1 year after BT might be related to the decrease in respiratory resistance during expiration (Table 1 and Fig. 1), and may have been affected by a reduction in the airway smooth muscle by BT, as demonstrated in multiple studies [12]. After BT, the sufficient exhalation obtained increased the time for inhalation, as shown by the increase in the inspiratory duty cycle (Ti/Ttot) (Fig. 2f) from resting to peak exercise, and shortened the time for the expiratory flow-volume curve to reach a nadir (Fig. 1). In general, the Ti/Ttot at rest is lower in asthmatics than in normal subjects [13, 14]; however, the exertional relationship between Ti/Ttot and dyspnea has not been studied completely. On the other hand, in patients with chronic obstructive pulmonary disease, we have confirmed that under similar ventilation conditions during exercise, the ability to absorb oxygen was higher when the Ti/Ttot increased than when the Ti/Ttot decreased [15]. Therefore, the increase in the ventilation equivalent for oxygen was suppressed during the late exercise phase (Fig. 2g); this implied that adequate ventilatory efficiency to absorb oxygen was obtained after BT. Laveneziana et al. [16] reported that the predominant exertional symptom in asthmatics was increased inspiratory effort, rather than expiratory effort, regardless of the presence of DH. Given the report, after BT in the present case, the longer inspiration time obtained by exhalation of enough trapped air may have led to effective and effortless pattern of breathing and reduction of the asthmatic symptoms during exercise (Fig. 2h).

There were some limitations of the present case study. First, although FOT might not be universally accepted, detailed analyses, including bronchial challenge test, should have been performed to confirm the presence of bronchial responsiveness before and after BT. Second, monoclonal antibody treatment prior to invasive BT should have been indicated in the present patient. However, in the light of the cost of prolonged asthma therapy [17], BT was chosen instead monoclonal antibody treatment for the present case. BT could become costly and therapeutically effective, if the mechanisms by which BT improves the symptoms of asthma are elucidated and if the patients who can respond to BT are identified.

## Conclusions

In the present case, BT did not improve the resting pulmonary function. However, after BT, V_Tex_ exceeded V_Tin_ form resting to peak exercise, which implied that a sufficient exhalation enabled longer inspiratory time, i.e., the higher Ti/Ttot, and the required ${\dot{\text{V}}}_{\text{E}}$ was reduced throughout exercise. Exertional dyspnea and exercise duration were primarily improved by obtaining better breathing patterns. Based on these, the capability of CPET to quantify the treatment effect demonstrated that breathing pattern may be an important mechanism of exertional dyspnea in asthmatics, which in turn might become a better predictor of response to BT. Further analyses, and lager studies are required to elucidate the clinical effectiveness of BT, with focus on breathing pattern in asthmatics.
